# Current News and Opinion

**Published:** 1883-11

**Authors:** 


### Current News and Opinion.

### "DIFFICULT DENTITION AND THE USE OF THE GUM LANCET."

[The author's proof corrections of the above entitled article did not arrive in time, and the printers could not wait, because of the absolute necessity for going to press with the first form. The following note, which was added, is therefore inserted here.—EDITOR.]

Dr. C. F. W. Bodecker, of this city, reported at the last meeting of the First District Dental Society a case which illustrates the subject, and of which he has kindly written the following account:

"A few days ago I saw a case with some very interesting reflex phenomena. The patient was a lady about twenty years of age, with a good constitution. When she entered my office she was suffering severely, so that she was hardly able to speak, and could only breathe with difficulty. The patient told me that for the last week whenever the paroxysm of pain came on she had experienced great difficulty in breathing, especially on the right side. On this side the first upper molar had been lately filled with amalgam, and ever since the filling had been introduced she had suffered excruciating pain. As the tooth was not sensitive to percussion, I diagnosed pulpitis, and perforated the pulp chamber. From the moment that the blood began to flow from the drill-hole the patient was relieved from pain, as well as from the dyspnœa, which up to this time has not recurred. I removed a part of the amalgam filling, which upon examination proved to be just the offending portion. It had been laid directly upon the exposed pulp, and a little eminence on this piece of filling showed that it had entered the pulp chamber. The pulp by the unskillful manner of filling had been irritated to such a degree that I found it dead the next day."

### A CLEAR DIAGNOSIS.

Incidents of a highly ludicrous nature frequently occur in the examination of patients before a class. A professor on one occasion was lecturing to his class on the means of diagnosing disease by the external appearance, face, and other details of the patient. Expressing his belief that a patient before the class afforded an example of the practice in question, the professor said to the indivdual: "Ah! you are troubled with gout!" "No, sir," said the man, "I've never had any such complaint!" "But," said the professor, "your father must have had gout?" "No sir," was the reply, "nor my mother either." "Ah, very strange," said the professor to his class. "I'm still convinced that this man is a gouty subject. I see that his front teeth show all the characters which we are accustomed to note in gout." "Front teeth," ejaculated the patient. "Well, that beats everything. It's the first time I've ever heard of false teeth having the gout. I've had this set for the last ten years." The effect of this sally, on the part of the patient, upon the inquisitorial professor and his students, may be better imagined than described.

---

### WOOD WOOL—A NEW SURGICAL DRESSING.

In Germany the reign of carbolic acid is over, and corrosive sublimate, or sublimate as it is there called, reigns in its stead. Many substances impregnated with sublimate, such as glass, wool, ashes, sand, etc., have been employed as attempts at permanent dressings with greater or less success. Something has still been wanting, something that will absorb a large quantity of discharges, and at the same time remain aseptic. Professor Bruns' (Tübingen) wood wool (*holswolla*) is finely ground wood, such as is used in the manufacture of paper. It is clean looking, delicate fibered, soft, yellowish-white, having an odor of fresh wood, and absorbs immensely.—*Excerpt from Medical Press.*

---

### HOT WATER IN THE TREATMENT OF INFLAMMATION OF MUCOUS MEMBRANES.

Dr. George R. Shepherd, Hartford, Conn., says:—"I have used hot water as a gargle for the past six or eight years. In acute pharyngitis and tonsillitis, and in coryza, or cold in the head, if properly used in the commencement of the attack, it constitutes one of our most effective remedies, being frequently promptly curative.

To be of service it should be used in considerable quantity, (a half pint or a pint at a time,) and just as hot as the throat will tolerate. I have seen many cases of acute disease thus aborted, and can commend the method with great confidence."

### IGNORANT TEACHERS.

"Odontalgia, I need not tell you, is one of the most common of painful affections, and every one has at some time experienced the atrocious pain of toothache. This neuralgia is often determined by *alveo-periostitis,* or by a carious tooth, which affects the terminal portion of the dental nerve. There exists a ready means of relief for this pain in arsenious acid, which destroys the dental pulp, a method which Tomes, Magitot and Combe have advised."—*From a clinical lecture by Prof. Dujardin-Beaumetz, Paris, France. Member of the Academy of Medicine; Physician to the Hospital St. Antoine, etc., etc., etc.*

### DEATH FOLLOWING EXTRACTION OF A TOOTH.

Two cases of death after extraction of teeth, from infection of the wound, are reported in *Wratsch.* Both were strong, healthy men. The reporter, Dr. Sacharewitsch, recommends the careful disinfection of instruments and hands, and the use of antiseptic washes, (carbolized water one to two per cent) after extraction of teeth. After bleeding has stopped, the wound may be packed with iodoform and sealed with cotton wadding dipped in collodion. The pain ceases almost at once, and no reaction occurred in nineteen cases so treated.

### DENTAL CONVENTION.

The Connecticut Valley Dental Society will hold its annual meeting at the Haynes House, Springfield, Mass., Nov. 7th and 8th, 1883.

The Members of the Society are requested to contribute material to their different Sections, so that when called, matter of interest and value may be reported.

Per Order of Executive Committee.

A. M. Ross, Secretary.

### BROOKLYN DENTAL SOCIETY.

The Brooklyn Dental Society held its annual meeting on Monday evening, October 8th, at the residence of Dr. J. H. Race, 366 Clinton Street.

The following were the officers elected for the ensuing year:

President—J. H. Race.

Vice President—J. B. Brown.

Recording Secretary—L. G. Wilder.

Corresponding Secretary—Will H. Johnston.

Treasurer—F. C. Walker.

Librarian—W. M. Ramsdell.

The society is in a prosperous and harmonious condition, and proposes to hold a place in the front ranks.

WILL H. JOHNSTON,
Cor. Secretary.

---

### A JUST CRITICISM.

Our journals have much to answer for to the profession, not the least of which is the charge of being largely instrumental in giving to some men a reputation for ability and representative standing that they never could have acquired by a strict reliance upon intellectual qualification. "One Journal," indeed! An almanac is more than sufficient for the caliber of some dental intelligencies.—*Extract from a rejected communication.*

---

### INSANITY IN THE UNITED STATES.

The tenth census gives some interesting and suggestive facts relative to the increase of insanity in this country. The total number of insane in 1870 was estimated at 37,432, as against 91,997 in 1880—an apparent increase of over 100 per cent. This gives a ratio of one insane person to every 543 of the population—a much larger estimate than many observers will be willing to admit.

